# Evaluation of the Stomatognathic System in Patients with Hearing Impairment and Cochlear Implants—A Pilot Study

**DOI:** 10.3390/jcm14165768

**Published:** 2025-08-14

**Authors:** Karolina Szuflak, Karolina Gerreth, Roksana Malak, Beata Wolnowska, Włodzimierz Samborski, Michał Karlik

**Affiliations:** 1 Department of Phoniatrics and Audiology, Poznan University of Medical Sciences, 60-355 Poznan, Poland; beata.wolnowska@ump.edu.pl (B.W.); mkarlik@ump.edu.pl (M.K.); 2 Doctoral School, Poznan University of Medical Sciences, 60-812 Poznan, Poland; 3 Department of Risk Group Dentistry, Chair of Pediatric Dentistry, Poznan University of Medical Sciences, 60-812 Poznan, Poland; karolinagerreth@ump.edu.pl; 4 Department and Clinic of Rheumatology, Rehabilitation and Internal Medicine, Poznan University of Medical Sciences, 61-545 Poznan, Poland; rmalak@ump.edu.pl (R.M.); wlodzimierz.samborski@ump.edu.pl (W.S.)

**Keywords:** Nordic Orofacial Test-Screening (NOT-S), orofacial function, oral health, parafunctions, dysfunctions, hearing loss

## Abstract

**Background:** Orofacial dysfunctions are a source of discontent and impair daily living activities. Patients with hearing impairments exhibit an elevated risk of stomatognathic system changes. Hence, this pilot study aims to evaluate changes in stomatognathic system functions in patients with hearing impairments and cochlear implants. **Methods**: During the examination, the Nordic Orofacial Test-Screening was used to assess orofacial functions. The range of motion within the temporomandibular joints was measured using a vernier caliper. A socio-medical study was conducted to collect data regarding the patients, the cochlear implant sides, and the methods of communication. **Results**: The statistical analysis showed a significant difference in the total NOT-S scores (*p* < 0.001) and examination scores (*p* < 0.001) between patients with hearing impairments and the control group. These significant differences of the total NOT-S score and examination score have large effect sizes (r > 0.5). In particular, significant differences were observed in the results of maximum mouth opening (*p* = 0.006) and right laterotrusion (*p* = 0.020). Differences were also observed in the answers regarding the method of communication and the examination score of the NOT-S questionnaire (*p* = 0.040). The 6A of the NOT-S significantly affected the examination score (*p* = 0.015) and the total NOT-S score (*p* = 0.037), while the result of section 6B only significantly affected the NOT-S examination score (*p* = 0.032). **Conclusions**: Patients with hearing impairments presented orofacial dysfunctions significantly more often than the control group. The side of cochlear implant implantation is important for stomatognathic system changes.

## 1. Introduction

Disorders within the stomatognathic system are frequently discussed in medical sciences [1,2,3,4,5,6]. The diagnosis and treatment of dysfunctions of the masticatory system is complex because the system’s proper functioning depends on the cooperation and mutual interactions of a set of tissues and organs of the oral cavity and the facial skeleton [6]. Activities related to the stomatognathic apparatus can be divided into physiological functions, dysfunctions, and parafunctional habits [7]. Proper physiological functions may be characterized through biting, chewing, swallowing, breathing, facial expressions, self-stimulation, coughing, and sneezing [8]. These actions comprise orofacial functions and result from the complex and integrated stimulation of the central nervous and neuromuscular systems [9]. It is worth noting that the proper operation of the stomatognathic system determines homeostasis between basic life functions and the expression of emotional states. The inability to perform these activities correctly can be identified as a dysfunction [10]. In turn, parafunctions are defined as unconsciously executed and regularly repeated incorrect actions [1]. The first type, short-circuit parafunctions, affects tooth connections and includes grinding (bruxism) during the day or night and clenching [11]. A scientific report in 2019 showed that the appearance of bruxism can be observed in 6–20% of the population, regardless of age, starting from the eruption of the deciduous teeth [1]. A meta-analysis from 2003 to 2023 indicated that the global sleep bruxism prevalence is 21% and awake bruxism prevalence is 23%. Moreover, sleep bruxism varies across continents; the lowest sleep bruxism prevalence is found in Asia at 19% and the highest in North America at 31%, while awake bruxism ranges from 18% in Europe to 30% in South America [12]. The other type of parafunction, non-short-circuit, occurs when the opposing teeth have no contact, e.g., biting nails or cuticles; thumb or lip sucking; biting on objects such as pencils, needles, or nails; and prolonged chewing of gum [11]. All these activities repeated many times over a long period can cause structural changes and ailments within the stomatognathic system and the head and neck area [13]. Due to the complexity of temporomandibular disorder diagnostics, the medical care of affected individuals should always be multidisciplinary.

Verbal communication is one of the main orofacial functions that can be disturbed in patients with hearing impairments [14,15,16,17]. According to the 2021 World Health Organization reports, hearing loss affects over 5% of the world’s population, representing approximately 430 million people. This number is expected to increase to 700 million by 2050, meaning that 1 in 10 people will experience hearing loss above 35 dB HL [18]. After analyzing the differences in the amount of language input between children with and without hearing loss, Sultana et al. indicated insufficient information on this subject [19]. While there are some reports about difficulties with speech production and intelligible speech within this group of patients [20], research is lacking regarding the diagnosis and possible causes of these dysfunctions.

In turn, there are some studies about the high prevalence of dental caries in patients with hearing impairments [14,15,16,19]. Kuroyedova and Sokolohorska-Nykina examined communication difficulties in hearing-impaired patients during dental visits [21]. There are also some reports about poor oral hygiene practices and periodontal status among adolescents with special needs and various disabilities [22,23]. As orofacial area problems and functional disturbances in such individuals are relatively common, supporting their oral health and stomatognathic apparatus should be prioritized.

There is an ongoing discussion regarding the potential influence of cochlear implants on orofacial function or stomatognathic system activities. Bicas et al. and Scarabello et al. suggested that cochlear implants positively impact the scores in the procedures that evaluate auditory and verbal development [24,25].

Moreover, there is a lack of publications concerning the association between orofacial dysfunctions and changes in the stomatognathic systems in patients with hearing impairments. There are also no data in the scientific literature on whether the side of an implanted cochlear implant influences the functions of the head and neck area. Hence, this study evaluated whether patients with hearing impairment may have more changes within the stomatognathic system than healthy individuals. To achieve this aim, we have assessed the changes in stomatognathic system activities in patients experiencing hearing impairment who have cochlear implants.

## 2. Materials and Methods

### 2.1. Materials

This study was approved by the Poznan University of Medical Sciences’ Bioethical Committee (resolutions 804/20, 482/21, and 874/22).

The examination was conducted from October 2022 to April 2023 with a group of 34 subjects (study group), 16 women (47.06%) and 18 men (52.94%), aged 6 to 44 years old (16.18 ± 10.11). All of the participants exhibited bilateral profound hearing loss and were implanted with cochlear implants. Twenty patients had cochlear implants on the right side (58.82%), seven had implants on the left side (20.59%), and seven had bilateral cochlear implants (20.59%). All patients were treated at the Department of Otolaryngology and the Department of Phoniatrics and Audiology, Poznan University of Medical Sciences, Poland. The control group was matched to the examination study group in terms of the number of individuals, age, and sex. The participants in the control group were recruited from among the patients and their parents from the University Center for Dentistry and Specialized Medicine in Poznan, Poland, as well as through advertisements posted in health centers and online. The directors of the institutions where the recruitment was conducted agreed to participate in this study. The inclusion and exclusion criteria are presented in Table 1. The sole inclusion criterion was the presence of profound hearing impairment and cochlear implant implantation. The exclusion criteria were the presence of facial trauma, nervous system disorders, genetic syndromes, and genetically determined diseases, as well as chronic diseases that could affect orofacial function. All subjects were asked if they had not had any problems or complaints related to temporomandibular disorders in the past. During all examination steps, the subjects under 18 years old or those who did not speak were accompanied by their legal guardians. Before participation, all participants or their guardians were informed about this study and expressed written informed consent.

### 2.2. Methods

Each examination step was conducted by the same person with a master’s degree in physiotherapy.

Socio-medical study questionnaires were handed over to adult participants or the guardians of the examined children. These included questions about the etiology, kind, and degree of hearing impairment, hearing aids, and cochlear implants, and communication methods.

#### 2.2.1. Orofacial Function Examination

The Nordic Orofacial Test-Screening (NOT-S) is a reliable and validated tool developed to screen and diagnose function in the orofacial region in patients older than three years of age [9]. NOT-S was created in 2007 by Bakke et al. and is recommended for diagnosing patients with speech, chewing, and swallowing difficulties and other orofacial dysfunctions. It consists of two parts, a structured interview which includes questions about (I) sensory function, (II) breathing, (III) habits, (IV) chewing and swallowing, (V) excessive salivation, and (VI) mouth dryness, as well as a clinical examination that assesses sections such as (1) the face at rest, (2) nasal breathing, (3) facial expression, (4) mandibular and masticatory muscle function, (5) oral motor function, and (6) speech. If one of the dysfunctions exists in a participant, the examiner notes one point. Each part contains six sections, so the final score is a sum that ranges from 0 to 12. Higher values indicate more difficulties in orofacial functions [9]. The examination part includes a picture manual to support patient understanding. The NOT-S questionnaire is available in many languages and can be downloaded from the Mun-H-Center website [26]. As this study was conducted with Polish patients, we have used a translated and validated Polish version of the questionnaire [27].

#### 2.2.2. Measurement Examination

The temporomandibular joint range of motion was evaluated using a vernier caliper measurement (DIGITAL CALLIPER No. HG00962A) [17,28,29,30,31,32]. The protrusion movement, maximum mouth opening, and laterotrusion movement on the right and left sides were assessed [31]. The protrusion between the incisors’ labial surface and the lower teeth’ lingual surface was measured. Maximum mouth opening was evaluated as the distance in the midline between the upper and lower incisors, while laterotrusion was designated by the distance between the midline of the maxilla and the mandible [29]. All measurements were obtained by verbally encouraging participants to perform the activity as far as possible. The results were corrected to 0.1 cm. The norm of the measures was established as a maximum mouth opening of above 40 mm for all subjects. As the minimum age was six years old [31], children were included in the same group as adults [33]. A shift norm in the mandibular midline was established from 7 ± 2 mm, and protrusive movement from 7 ± 2 mm [31,34].

#### 2.2.3. Socio-Medical Study

Adult subjects or guardians of children, before the examination, answered the socio-medical questionnaire. The questions were, “On which side is the cochlear implant?”, “When was it implanted?”, and “What is the method of communication of the patient?” with possible answers of “Creates complex sentences”, “Does not speak”, or “Uses simple words”.

### 2.3. Statistical Analysis

The data obtained were subjected to statistical analysis using the PQStat v.1.8.4. software. The Mann–Whitney U-Test was used to determine statistically significant results among the following: total NOT-S, interview, examination scores, and range of motion measurements between the examination and control groups, and to establish dependence between answers from a socio-medical study, the NOT-S scores. The chi-square test was used to assess the associations between the results of each section of the NOT-S questionnaire. The effect size for a Mann–Whitney U test was calculated using r = Z/√n, where Z is the standardized test statistic, and n is the total sample size [35]. The interpretation of r was as follows: r < 0.3 indicated a small effect, indicating a weak difference between the groups; a result between 0.3 ≤ r < 0.5 was considered a medium effect, suggesting a moderate difference; and r ≥ 0.5 was considered a large effect [36].

Results with *p* ≤ 0.05 were considered statistically significant.

## 3. Results

The results showed that only two subjects from the study group (5.88% of the total) obtained a total NOT-S score of 0.0, indicating they had no orofacial dysfunctions (Table 2). For comparison, in the control group, such a score was observed in 13 subjects (38.24% of the total). The statistical analysis showed a significant difference (*p* < 0.001) between the total NOT-S score in the study group and the control group, with the results amounting to 7.0 and 4.0, respectively (Table 2). In addition, the presence of a measurement examination score of 0.0 also showed a difference (*p* < 0.001) between hearing impairment in the participants (12 subjects; 35.29%) and the control group (31 subjects; 91.18%). These significant differences of the total NOT-S score and the examination score have a large effect size (r > 0.5) (Table 2).

The statistical analysis indicated no significant differences between the study group and the control group among the sections and the questions of the NOT-S questionnaire (Table 3). Nonetheless, the results demonstrate that the most common orofacial dysfunctions in the study group were problems with speech, which concerned as many as 17 subjects (50% of the total) (Table 3).

The range of motion results of the temporomandibular joints show that all measurements were lower in the study group than in the controls (Table 4). Statistically significant differences were observed in comparison between the groups regarding the results of the maximum mouth opening (*p* = 0.006) and laterotrusion right (*p* = 0.020). These significant results had a medium effect size (0.3 ≤ r < 0.5). The average result of the maximum mouth opening in the study group was 39.9 mm (median = 40, SD = 7.79) compared with 45.8 mm (median = 46, SD = 7.40) in the control group. The average right laterotrusion was 9.3 mm (median = 10, SD = 3.06) in subjects with hearing impairment, in comparison with the healthy participants, for whom the right laterotrusion amounted to 10.9 mm (median = 11, SD = 2.56) (Table 4).

Table 5 shows the results regarding the communication method (answers from a socio-medical study) and the NOT-S scores (total NOT-S score, interview score, and examination score) for patients with hearing impairments. Statistically significant results (*p* = 0.040) were observed for the question, “What is the method of communication of the patient?” Twenty-nine respondents marked “Creates complex sentences” and five subjects marked “Does not speak” or “Uses simple words” (Table 5). Three patients in the 6A section were assessed as “Does not speak,” with the result significantly affecting examination scores (*p* = 0.015) and the total NOT-S score (*p* = 0.037). In addition, significance was observed (*p* = 0.032) in the 6B section examination score of the NOT-s questionnaire —“Count out loud to ten”—which was not possible for 14 participants. The above significance results had a medium effect size (0.3 ≤ r < 0.5) (Table 5).

## 4. Discussion

This study demonstrates an association between changes in stomatognathic system activities in patients with hearing impairments with cochlear implants. We found a significantly higher occurrence of advanced orofacial dysfunctions within the hearing-impaired patients compared with the control group. The total NOT-S score and examination score were higher in the study group. The effect size of these results was large, so it suggests that it is meaningful and is not just due to random chance. Moreover, the range of motion regarding the right laterotrusion and maximum mouth opening was significantly limited in the study patients. In addition, the data suggest that patients who did not speak or formed only simple words could have a higher risk of problems with orofacial functions.

This study is the first project to assess orofacial functions of this complexity on an extensive scale among patients with hearing loss and cochlear implants. Our main aim was to compare the study and control groups using a NOT-S questionnaire. Significant results were obtained for the total NOT-S and measurement examination scores. The total NOT-S score in the study group ranged from 0.0 to 7.0, while the norm for the total NOT-S score of the healthy people was 2.0 [9]. In our study, 17 hearing-impaired patients (50% of the total) scored above the norm, which could indicate that this group of patients is at an elevated risk of orofacial dysfunctions. This confirms the results of a study by Shah et al., which had 70 deaf subjects (male 42, female 28, ages 12–18 years) [17]. The authors used Fonseca’s Questionnaire to assess temporomandibular disorders and a vernier caliper to measure the range of motion of the temporomandibular joints. The results showed that 23 (33% of the total) deaf participants had temporomandibular dysfunctions, and 33% of the subjects had decreased degrees of mouth opening [17]. One argument of the above study was that several deaf patients exhibited a complete lack of speech, which could have resulted in orofacial disorders. This argument also finds confirmation in our research, as the total NOT-S and measurement examination scores were significantly affected by the “Does not speak” answer in the 6A section (8.82% of the participants). This result emphasizes that speaking ability is essential for that group of patients, further supporting the value of cochlear implants.

In our examination, one of the statistically significant results is the measurement of right laterotrusion. The range of motion on the left side was closer to that of the control group. In previous studies, Coelho-Ferraz et al. and Korbmacher et al. indicated that asymmetrical muscle tension distribution could influence temporomandibular disorders [37,38]. This suggests that special attention should be dedicated to the range of motion and muscle tension around the head and neck in hearing-impaired patients after cochlear implantation.

These findings have clinical implications, suggesting that specialists refer patients to physiotherapists and conduct orofacial rehabilitation to improve the limited maximum mouth opening or to equalize the muscle tension if laterotrusion is in disbalance. Such a therapeutic approach could improve patients’ verbal communication, breathing, and nutrition. Acknowledging this study’s limitations, including the limited number of subjects, is essential. Nonetheless, this study is the first such examination of hearing-impaired patients with cochlear implants, so it can be considered a pilot study to motivate further projects regarding orofacial function screening in this group of patients. It would be helpful to compare two groups with and without cochlear implants, preferably in a similar age range. However, this could prove difficult, as for the last 23 years in Poland, there has been a Nationwide Programme for Newborn Hearing Screening that promotes the early detection of hearing loss, so the majority of patients with hearing loss are implanted with cochlear implants at a very young age [39]. This is the first time NOT-S has been used for patients with hearing problems, and it can be concluded that the picture manual, which is attached to this test, is a highly useful tool in explaining the expected action to be performed by patients.

To summarize, the results of our study show that patients with hearing impairments who have cochlear implants exhibited changes within their stomatognathic system activity, such as a limited range of motion in the temporomandibular joints. Moreover, orofacial dysfunctions were more common in the study group than in the control group. Interestingly, the side of implantation significantly impacted the results of the conducted examinations. These results indicate that a referral to specialists for rehabilitating orofacial functions, such as speech therapists and physiotherapists, could significantly improve the quality of life of patients with hearing impairments.

## 5. Conclusions

The results of our study contribute to a clearer understanding of the meaning of orofacial dysfunctions among patients with hearing impairments and cochlear implants. Such patients presented changes within their stomatognathic systems more often than the control group. Furthermore, the side of cochlear implantation is important for stomatognathic apparatus activity. These results should be considered in interdisciplinary and comprehensive treatment to improve speech rehabilitation.

## Figures and Tables

**Table 1:** Inclusion and exclusion criteria for the subjects in the study group.

Inclusion Criteria	Exclusion Criteria
hearing-impaired patients	patients with good hearing
implanted cochlear implant	patients with hearing impairment without a cochlear implant
no disorders of the nervous system	disorders of the nervous system in medical history
no genetic syndromes or genetically determined diseases that could affect orofacial function	participants with genetic syndromes or genetically determined diseases that could affect orofacial function
patients understood the orders	patients who cannot understand the instructions
patients were able to cooperate	patients without the will to cooperate
no craniofacial injuries in medical history	previous craniofacial injuries
participants or their guardians gave informed consent to this study	no informed consent to the study
conscious persons	unconscious persons

**Table 2:** Baseline characteristics of the participants within the study group and the control group.

Sample Characteristics		Study Group	Control Group	*p*-Value	ES
Sample count		n (%)	n (%)		
Sex	female	16 (47.06)	16 (47.06)		
	male	18 (52.94)	18 (52.94)		
V-age	Q1	9.25	9.25		
	Q2	21.5	21.5		
	mean	16.18	16.18		
	SD	10.11	10.14		
Answers with third-person assistance		25 (73.53)	23 (67.65)		
Total NOT-S score	0.0	2 (5.88)	13 (38.24)	<0.001 *	r = 0.63
	1.0	9 (26.47)	8 (23.53)		
	2.0	6 (17.65)	9 (26.47)		
	3.0	10 (29.41)	3 (8.82)		
	4.0	5 (14.71)	1 (2.94)		
	5.0	1 (2.94)	-		
	6.0	-	-		
	7.0	1 (2.94)	-		
Interview score	0.0	8 (23.53)	13 (38.23)	0.155	r = 0.24
	1.0	11 (32.35)	10 (29.41)		
	2.0	8 (23.53)	8 (23.53)		
	3.0	7 (20.59)	2 (5.88)		
	4.0	-	1 (2.94)		
Measurement examination score	0.0	12 (35.29)	31 (91.18)	<0.001 *	r = 0.83
	1.0	12 (35.29)	3 (8.82)		
	2.0	8 (23.53)	-		
	3.0	1 (2.94)	-		
	4.0	1 (2.94)	-		

- score not observed; * statistically significant result; significance level *p* ≤ 0.05; the chi-square test was used; n—number; %—percentage; Q3—upper quartile; Q1—lower quartile; ES—effect size; r < 0.3: small effect; 0.3 ≤ r < 0.5: medium effect; r ≥ 0.5: large effect.

**Table 3:** Distribution of NOT-S scores in each section and question.

Part	Name of Section	Number of Subsection	Study Group		Control Group		*p*-Value
			Each Question n (%)	Each Section n (%)	Each Question n (%)	Each Section n (%)	
Interview	I Sensory function	I A	1 (2.94)	2 (5.88)	0	3 (8.82)	0.650
		I B	1 (2.94)		3 (8.82)		
	II Breathing	II A	0	5 (14.71)	0	3 (8.82)	-
		II B	5 (14.71)		3 (8.82)		0.340
	III Habits	III A	13 (38.24)	17 (50)	10 (29.41)	18 (52.94)	0.491
		III B	6 (17.65)		11 (32.35)		
		III C	6 (17.65)		6 (17.65)		
	IV Chewing and swallowing	IVA	0	11 (32.35)	0	7 (20.59)	0.810
		IV B	8 (23.53)		4 (11.77)		
		IV C	3 (8.82)		0		
		IV D	1 (2.94)		4 (11.77)		
		IV E	1 (2.94)		1 (2.94)		
	V Drooling	V A	3 (8.82)	3 (8.82)	1 (2.94)	1 (2.94)	0.752
	VI Dryness of the mouth	VI A	8 (23.53)	8 (23.53)	4 (11.77)	4 (11.77)	0.183
		VI B	0		0		
Examination	1 Face at rest	1 A	0	1 (2.94)	0	0	-
		1 B	0		0		
		1 C	0		0		
		1 D	1 (2.94)		0		
	2 Nose breathing,	2 A	1 (2.94)	1 (2.94)	0	0	-
	3 Facial expression	3 A	0	5 (14.71)	0	0	-
		3 B	3 (8.82)		0		
		3 C	2 (5.88)		0		
	4 Masticatory muscle and jaw function	4 A	5 (14.71)	6 (17.65)	1 (2.94)	1 (2.94)	0.638
		4 B	1 (2.94)		0		
	5 Oral motor function	5 A	2 (5.88)	4 (11.77)	0	0	-
		5 B	1 (2.94)		0		
		5 C	1 (2.94)		0		
		5 D	0		0		
	6 Speech	6 A	3 (8.82)	17 (50)	0	2 (5.88)	0.144
		6 B	14 (41.18)		2 (5.88)		
		6C	4 (11.77)		0		

Significance level *p* ≤ 0.05; the chi-square test was used; n—number; %—percentage.

**Table 4:** Comparison of the results of the range of motion between the study group and the control group.

Sample Characteristics	Study Group					Control Group						
Sample Count	$\bar{x}$	Q1	Q3	min	max	$\bar{x}$	Q1	Q3	min	max	*p*-Value	ES
Protrusion	6.1	5	7	0	16	6.8	5	8.75	0	13	0.136	r = 0.26
Left laterotrusion	9.7	9	11.75	0	14	10.1	9	11.75	4	15	0.569	r = 0.10
Right laterotrusion	9.3	7.25	11	2	15	10.9	10	12	5	15	0.021 *	r = 0.40
Maximum mouth opening	39.9	35.5	45	20	55	45.8	40.25	51.75	36	60	0.006 *	r = 0.47

$\bar{x}$—average; Q3—upper quartile; Q1—lower quartile; * statistically significant result; significance level *p* ≤ 0.05; the Mann–Whitney U-Test was used. All measures are in millimeters (mm). ES—effect size; r < 0.3: small effect; 0.3 ≤ r < 0.5: medium effect; r ≥ 0.5: large effect.

**Table 5:** Associations among the kinds of communication (answers from a socio-medical study) and the NOT-S scores.

Sample Characteristics	n (%)	Total NOT-S Score		Interview Score		Examination Score	
		*p*-Value	ES	*p*-Value	ES	*p*-Value	ES
Answer from the socio-medical study “Creates complex sentences”	29 (85.29)	0.317	r = 0.17	0.999	r = 0	0.040 *	r = 0.35
Answer from the socio-medical study “Does not speak” or “Uses simple words”	5 (14.71)						
Point in NOT-S section 6A “Does not speak”	3 (8.82)	0.036 *	r = 0.36	0.330	r = 0.17	0.015 *	r = 0.42
Point in NOT-S section 6B “Count out loud to ten”	14 (41.18)	0.829	r = 0.03	0.079	r = 0.30	0.032 *	r = 0.36

* statistically significant result; significance level *p* ≤ 0.05; the Mann Whitney U-Test was used; ES—effect size; r < 0.3: small effect; 0.3 ≤ r < 0.5: medium effect; r ≥ 0.5: large effect.

## Data Availability

This article encompasses the original contributions outlined in this study. Additional inquiries may be sent to the corresponding author.

## Abbreviations

The following abbreviations are used in this manuscript:

NOT-S	Nordic Orofacial Test-Screening
